# 3-Amidinophenylalanine-Derived Inhibitors’ Antiviral Effect Against H1N1 Influenza A Virus

**DOI:** 10.3390/antibiotics15040366

**Published:** 2026-04-02

**Authors:** Lilla Tóth, András Marosi, Luna C. Schmacke, Torsten Steinmetzer, Anita Rácz, Dávid Bajusz, Ákos Jerzsele, Sándor Kunsági-Máté, Miklós Poór, Erzsébet Pászti-Gere

**Affiliations:** 1 Department of Pharmacology and Toxicology, University of Veterinary Medicine, István utca 2, H-1078 Budapest, Hungary; toth.lilla@student.univet.hu (L.T.); jerzsele.akos@univet.hu (Á.J.); 2 Virology Research Group, Department of Microbiology and Infectious Diseases, University of Veterinary Medicine, Hungária krt 23, H-1143 Budapest, Hungary; 3 Institute of Pharmaceutical Chemistry, Department of Pharmacy, Philipps University Marburg, Marbacher Weg 6-10, 35037 Marburg, Germany; luna.schmacke@pharmazie.uni-marburg.de (L.C.S.); torsten.steinmetzer@staff.uni-marburg.de (T.S.); 4 Plasma Chemistry Research Group, HUN-REN Research Centre for Natural Sciences, Magyar tudósok krt. 2, H-1117 Budapest, Hungary; racz.anita@ttk.hu; 5 Medicinal Chemistry Research Group and Drug Innovation Centre, HUN-REN Research Centre for Natural Sciences, Magyar tudósok krt. 2, H-1117 Budapest, Hungary; bajusz.david@ttk.hu; 6 National Laboratory of Infectious Animal Diseases, Antimicrobial Resistance, Veterinary Public Health and Food Chain Safety, University of Veterinary Medicine, István utca 2, H-1078 Budapest, Hungary; 7 Department of Organic and Medicinal Chemistry, Faculty of Pharmacy, University of Pécs, Honvéd u. 1, H-7624 Pécs, Hungary; kunsagi-mate.sandor@gytk.pte.hu; 8 Green Chemistry Research Group, János Szentágothai Research Centre, University of Pécs, Ifjúság útja 20, H-7624 Pécs, Hungary; 9 Department of Laboratory Medicine, Medical School, University of Pécs, Ifjúság útja 13, H-7624 Pécs, Hungary; poor.miklos@pte.hu; 10 Molecular Medicine Research Group, János Szentágothai Research Centre, University of Pécs, Ifjúság útja 20, H-7624 Pécs, Hungary

**Keywords:** TMPRSS2, matriptase, H1N1, 3-amidinophenylalanine, inhibitors, CYP3A, plasma protein binding, thrombin, proteolytic cleavage

## Abstract

**Background/Objectives:** Transmembrane serine proteases such as TMPRSS2 and matriptase have been identified as pivotal host factors in the influenza A infection due to their capacity to cleave the hemagglutinin and thereby facilitate viral activation. The inhibition of these enzymes has the potential to serve as an effective therapeutic strategy against numerous seasonal influenza strains. In our study, four 3-amidinophenylalanine-derived inhibitors were used to elucidate their antiviral efficacy, pharmacokinetic properties and affinities toward certain related trypsin-like serine proteases. **Methods:** K_i_ values for TMPRSS2, matriptase, thrombin and factor Xa were determined using enzyme kinetic measurements. Cytochrome P450 3A (CYP3A) inhibitory activity was investigated using human liver microsomes, and protein binding was evaluated with human serum albumin and α_1_-acid glycoprotein. *In vitro* antiviral efficacy and cytotoxicity were determined in MDCK-II cells. **Results:** All compounds were non-cytotoxic and exhibited a relatively high affinity toward matriptase and bovine thrombin in the 10–30 nM concentration range. Among the inhibitors, MI-441 displayed the lowest K_i_ value for TMPRSS2 (~60 nM). The weakest CYP3A inhibitory activity was observed for compounds MI-447 and MI-448. In addition, three of the four compounds (MI-441, MI-443 and MI-447) demonstrated significant antiviral activity. **Conclusions:** This study demonstrates that the investigated inhibitors exhibit a favorable safety profile, low binding to human serum albumin and pronounced antiviral activity against H1N1.

## 1. Introduction

The global burden imposed by re-emerging respiratory viruses—such as severe acute respiratory syndrome coronaviruses (SARS-CoV, SARS-CoV-2), Middle East respiratory syndrome coronavirus (MERS-CoV), and various subtypes of influenza A virus—has underscored the urgent need for novel antiviral strategies [1]. Traditionally, antiviral drug development has focused primarily on targeting viral proteins [2]. However, the high mutation rates of viruses often lead to rapid resistance development, rendering many of these approaches transiently effective. In contrast, the host-targeted antiviral strategies, especially those interfering with virus–host interactions required for viral entry, offer a promising and potentially more resistance-resilient alternative [3,4,5,6,7,8].

The proteolytic activation of viral surface glycoproteins by host serine proteases is a key factor in infectivity, tissue tropism, and pathogenesis for many respiratory viruses, including influenza A subtypes and coronaviruses such as SARS-CoV-2. Among these host factors, transmembrane protease serine 2 (TMPRSS2) has emerged as a critical enzyme for mediating spike (S) protein priming in SARS-CoV-2 and hemagglutinin (HA) cleavage in seasonal influenza viruses, thus enabling membrane fusion and viral entry [9,10,11,12,13,14,15]. TMPRSS2 is predominantly expressed in the epithelial cells of the upper and lower respiratory tract, with especially high expression in nasal, bronchial, and alveolar epithelial cells [15].

Despite its prominent expression pattern, genetic ablation of TMPRSS2 in mice results in no major developmental or physiological defects, suggesting that pharmacological inhibition is unlikely to cause significant toxicity [16]. The recent animal studies have also demonstrated the safety of *TMPRSS2* knockout (KO) models and provided essential information about the role of TMPRSS2 in influenza infection *in vivo.* Sakai et al. showed that *TMPRSS2* KO mice were tolerant against H1N1, H3N2, and H7N9 strains in contrast to wild-type (WT) animals [17]. Furthermore, *TMPRSS2* knockout mice were only protected from infections by influenza A strains, but not from influenza B strains [18]. The studies by Ciacci Zanella et al. and Kwon et al. revealed that pigs lacking *TMPRSS2* exhibited reduced inflammatory responses and fewer lung lesions when challenged with H1N1 and H3N2 influenza A strains [19,20].

Bestle et al. have found that both TMPRSS2 and furin are required for the efficient activation of the SARS-CoV-2 spike protein in human airway epithelial cells [9]. This proteolytic cascade presents multiple points for therapeutic intervention, and the combined inhibition of these enzymes could synergistically enhance antiviral efficacy. Besides TMPRSS2 and furin, human airway trypsin-like protease (HAT) has also been recognized as a protease involved in the activation of various influenza and coronaviruses [21,22,23,24]. The SARS-CoV-2 entry into host cells is mediated by the angiotensin-converting enzyme 2 (ACE2), which acts as the host cell receptor, and this process can be inhibited by TMPRSS2 inhibitors, indicating the protease’s essential role in spike protein priming [11].

Matriptase also plays either a redundant or complementary role to TMPRSS2 in activating certain viral glycoproteins, particularly in influenza A viruses, including H1N1, H9N2, and H7N9 strains [21,25,26]. The enzymatic profiling and kinetic studies have shown that many TMPRSS2 inhibitors, particularly ketobenzothiazole (KBT) derivatives, such as MM3122 and N-0100, display a significant cross-reactivity with matriptase, which may enhance their broad-spectrum antiviral potential [26,27].

As demonstrated in previous studies, a range of arylsulfonylated 3-amidinophenylalanine (Phe(3-Am)-)-piperidide derivatives have been shown to possess potent inhibitory activity against multiple type II transmembrane serine proteases (TTSPs) [21,28,29,30], and some of them possess significant antiviral effects against various strains of both coronaviruses and influenza viruses [9,31]. These compounds represent structurally related analogs from a previously described Phe(3-Am)-based inhibitor series, which were selected to evaluate how variations in the N-terminal biphenyl sulfonyl substitution pattern and in the C-terminal tertiary amide moiety influence TMPRSS2/matriptase inhibitory potency and antiviral activity.

**Figure 1:**
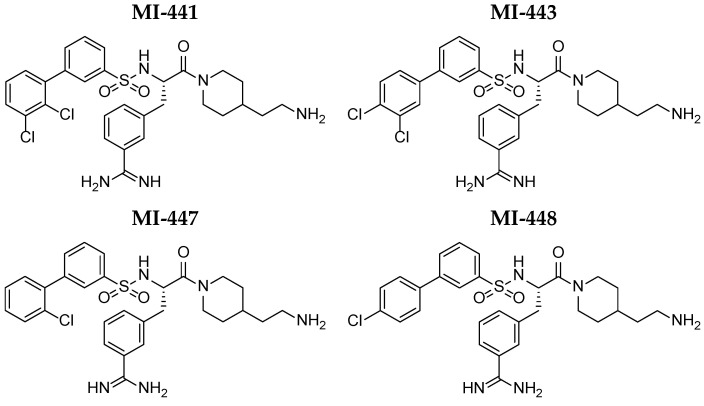
The structures of the used 3-amidinophenylalanine-derived inhibitors. The structures of MI-441 and MI-443 were determined by Hammami et al. (2012) [29], and the inhibitor compounds MI-447 and MI-448 were determined by Steinmetzer et al. (2009) [28].

In this study, the pharmacodynamic (PD) profile of four Phe(3-Am)-derived inhibitors (MI-441, MI-443, MI-447, and MI-448; see Figure 1) was determined, focusing on antiviral potency and certain pharmacokinetic (PK) parameters. We investigated target selectivity in order to predict the potential bleeding risk associated with off-target interactions with coagulation-related proteases. The inhibitor molecules’ antiviral effect on the H1N1 influenza virus was also assessed.

## 2. Results

### 2.1. Determination of the Inhibitors’ K_i_ Values

The inhibition constants (K_i_) for matriptase ranged from 11 to 29 nM. The compound MI-441 (Figure 1) exhibited the most potent inhibitory activity (K_i_ ≈ 11 nM). In addition, bovine thrombin (b-thrombin) was inhibited with similar K_i_ values; the compounds MI-447 (K_i_ = 14 nM) and MI-448 (K_i_ = 15 nM) were identified as the most potent thrombin inhibitors in this assay (Table 1).

The inhibition of fXa was substantially weaker; all the determined K_i_ values were >260 nM. The moderate inhibition of TMPRSS2 was observed across the series. Consistent with its strong matriptase inhibition, compound MI-441 demonstrated the lowest K_i_ value for TMPRSS2 (63 ± 5 nM) while other compounds (MI-443, MI-447, and MI-448) exhibited a weaker TMPRSS2 inhibition, with inhibition constants ranging from ~200 to 400 nM (Table 1).

**Figure 2:**
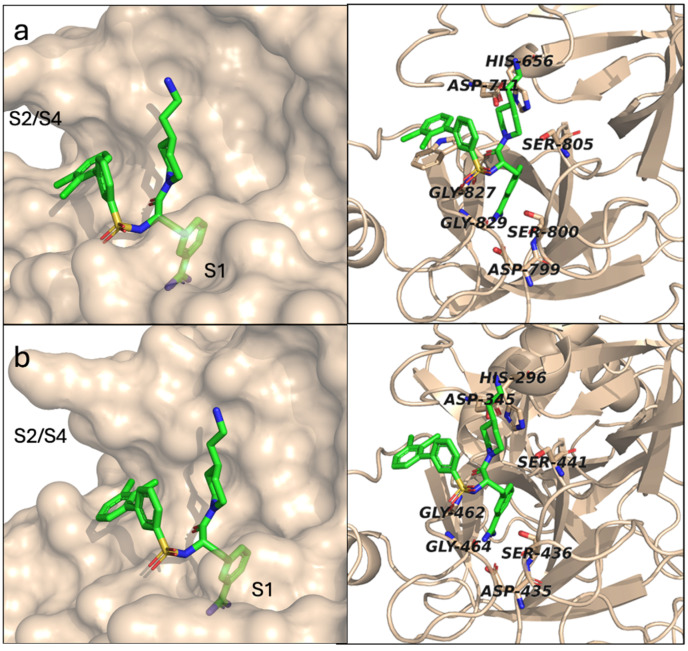
The predicted binding modes of MI-441 in the active sites of matriptase (**a**) and TMPRSS2 (**b**). The secondary interactions are shown with dashed lines (yellow: H-bond, magenta: salt bridge). The binding site regions and residues with notable interactions (with chymotrypsinogen numbering) are labeled.

To establish a structural basis for the observed inhibitory activities, we have predicted the binding mode of MI-441 (as the most potent inhibitor of the series) in matriptase and TMPRSS2 with Chai-1, using a state-of-the-art co-folding model [32]. Reassuringly, MI-441 was placed in the respective active site, exhibiting the “canonical” binding pose of this family of protease ligands with its arginine mimetic group (amidine) establishing a key salt bridge with the Asp189 residue of the S1 subpocket, supported by further H-bonds to the neighboring Ser190 and Gly219 residues, as well as an alternating, acceptor–donor–acceptor pattern of H-bonds to the backbone atoms of Gly216 and Gly219 (Figure 2, the residue positions refer to the chymotrypsinogen numbering).

### 2.2. Evaluation of CYP3A Inhibition

**Figure 3:**
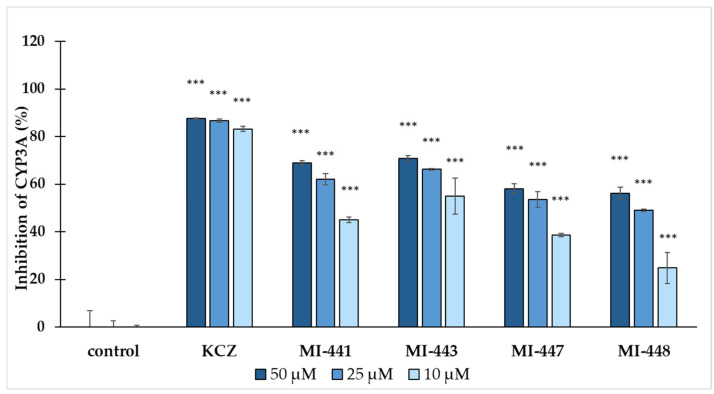
The CYP3A inhibition. The effects of the tested inhibitors and the positive control ketoconazole (KCZ) on the CYP3A activity were assessed at concentrations of 10 µM, 25 µM, and 50 µM. The average CYP3A inhibition percentage values ± SDs were expressed relative to the control levels. All the compounds demonstrated a statistically significant inhibition of CYP3A activity at each tested concentration (*** *p* < 0.001; n = 3).

The inhibitory potential of the antiviral compounds on the CYP3A activity was evaluated using human hepatocyte microsomes with ketoconazole (KCZ) included as a positive control (Figure 3). All the tested compounds demonstrated a significant inhibition of the CYP3A activity compared to the untreated control group (in the absence of inhibitors), including at the lowest tested concentration of 10 µM (*** *p* < 0.001).

Among them, the compound MI-448 consistently demonstrated the weakest CYP3A inhibition across all the tested concentrations (Figure 3), suggesting the lowest likelihood of CYP3A-mediated drug interactions. The inhibitor MI-447 exhibited a similarly low inhibitory capacity. In contrast, MI-443 showed the strongest inhibitory activity at all concentrations, and the differences between the compounds MI-443 and MI-448 were statistically significant at every tested concentration point (*** *p* < 0.001).

### 2.3. Assessment of the Interactions with HSA and AGP Based on Fluorescence Spectroscopic Studies

**Figure 4:**
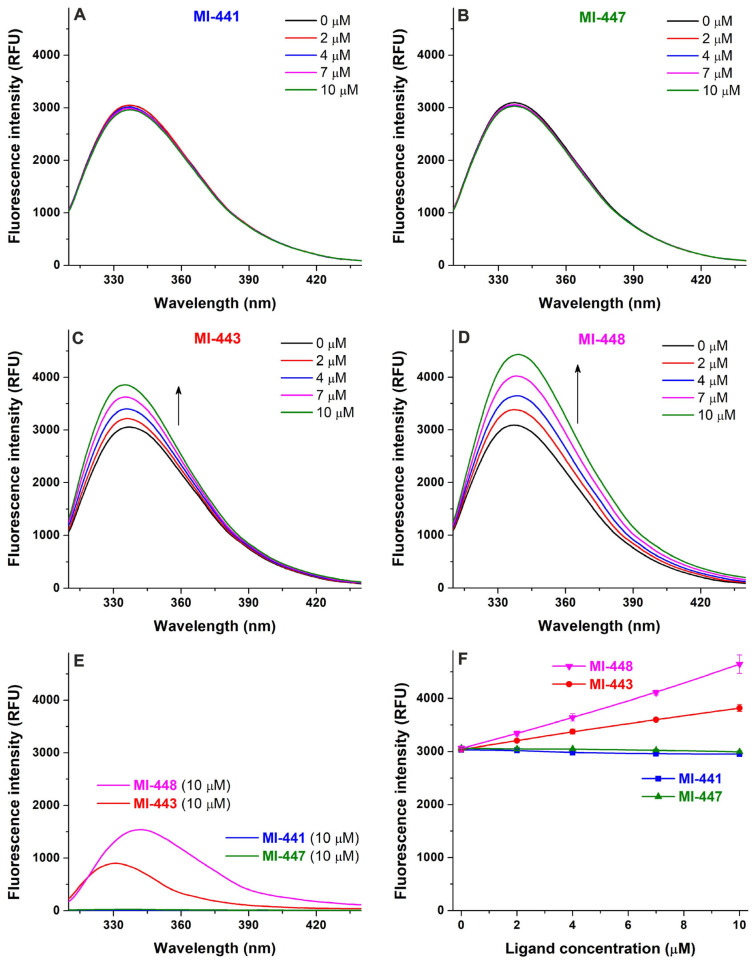
The fluorescence emission spectrum of the HSA (2 μM) with and without increasing concentrations (0–10 μM) of inhibitors MI-441 (**A**), MI-447 (**B**), MI-443 (**C**), and MI-448 (**D**) in PBS (pH 7.4; λ_ex_ = 295 nm). The fluorescence background signals of MI-441, MI-443, MI-447, and MI-448 (**E**). The concentration-dependent changes that occurred in the fluorescence emission intensity at 340 nm (**F**). The background and the inner-filter effect have been corrected; see UV spectra of MI compounds in Figure S1.

The inhibitors MI-441 and MI-447 did not affect the emission signal of HSA (Figure 4A,B). Although compounds MI-443 and MI-448 induced linear elevation in the emission intensities at 340 nm (Figure 4C,D), it was a direct result of their intrinsic fluorescence at this wavelength (Figure 4E,F). Thus, derivatives MI-443 and MI-448 did not change the emission signal of HSA. These observations suggest no relevant interactions of the four ligands examined with the HSA.

**Figure 5:**
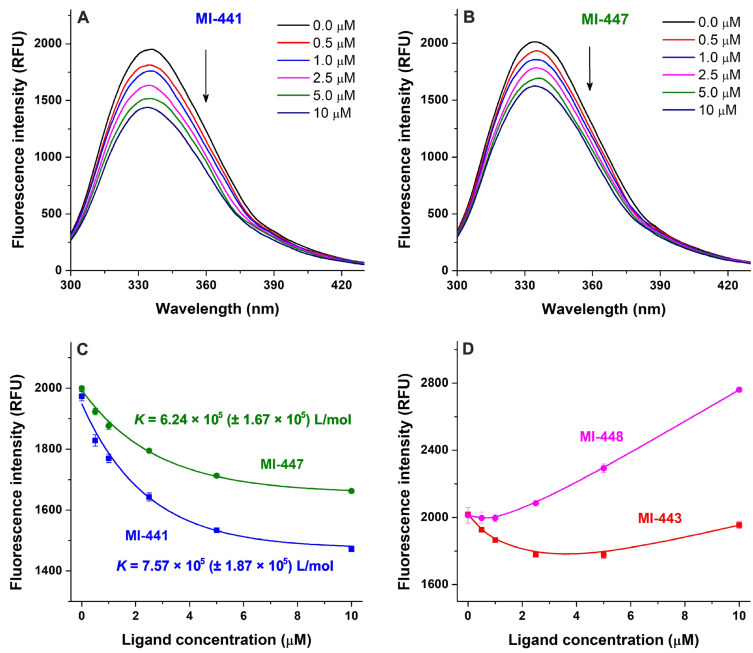
The fluorescence emission spectrum of AGP (2 μM) with and without increasing concentrations (0–10 μM) of inhibitors MI-441 (**A**) and MI-447 (**B**) in PBS (pH 7.4; λ_ex_ = 285 nm; see data for MI-443 and MI-448 in Figure S1). The absorption spectra and fluorescence background signals are demonstrated in Figure S1. The concentration-dependent changes in the fluorescence emission intensity at 335 nm in the presence of MI-441 and MI-447 (**C**), and MI-443 and MI-448 (**D**), with the background and the inner-filter effects corrected. The calculated binding/association constants (*K_a_* ± SD) for MI-441–AGP and MI-447–AGP complexes are also indicated.

Compounds MI-441 and MI-447 induced concentration-dependent decreases in the emission signal of AGP (Figure 5A,B), showing the saturation-type concentration–intensity curves (Figure 5C). The lower concentrations (0.5–2.5 μM) of MI-443 gradually reduced the fluorescence at 335 nm, after which the signal was elevated in the presence of 10 μM ligand concentration (Figure 5D and Figure S1). Furthermore, 0.5 and 1.0 μM of the inhibitor MI-448 resulted in negligible changes in the emission signal of AGP, while higher ligand concentrations (e.g., 5 and 10 μM) led to considerable increases at 335 nm (Figure 5D and Figure S1). The complex changes caused by MI-443 and MI-448 can be explained by two competing effects with opposite directions: (1) the complex formation of these ligands with AGP reduces the emission signal of the protein at 335 nm, and (2) both MI-443 and MI-448 exert significant fluorescence at this wavelength (Figure S1), which causes an elevation in the emission intensity. At lower ligand concentrations (around 0.5–2.5 μM), the quenching effect dominates (MI-443) or at least it compensates (MI-448) the emission signals of the ligands added. However, in the presence of higher ligand concentrations (5 and/or 10 μM), the interactions of MI-443 and MI-448 with AGP likely approximate the saturation (as it has been demonstrated for MI-441 and MI-447 in Figure 5C); therefore, the fluorescence signal of the ligands dominates the quenching-induced decreases. Since MI-441 and MI-447 have no relevant background signals at 335 nm (Figure S1), we estimated the binding/association constants (*K_a_*) of their AGP complexes. Based on the non-linear fitting with the HypSpec 2014 software, we assume the formation of 1:1 stoichiometry complexes with *K* values of 7.57 × 10^5^ L/mol for MI-441–AGP and 6.24 × 10^5^ L/mol for MI-447–AGP.

### 2.4. Antiviral Effect of Inhibitors on H1N1 Influenza Virus Replication

**Figure 6:**
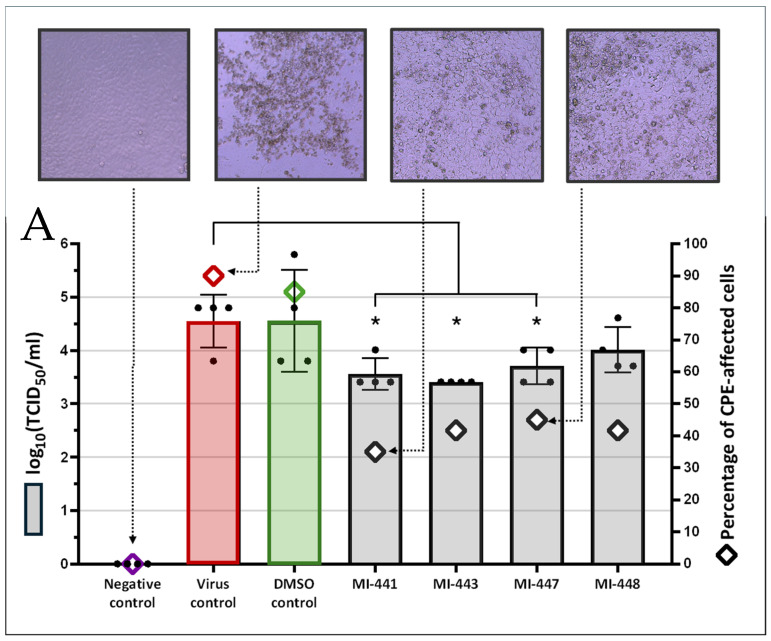

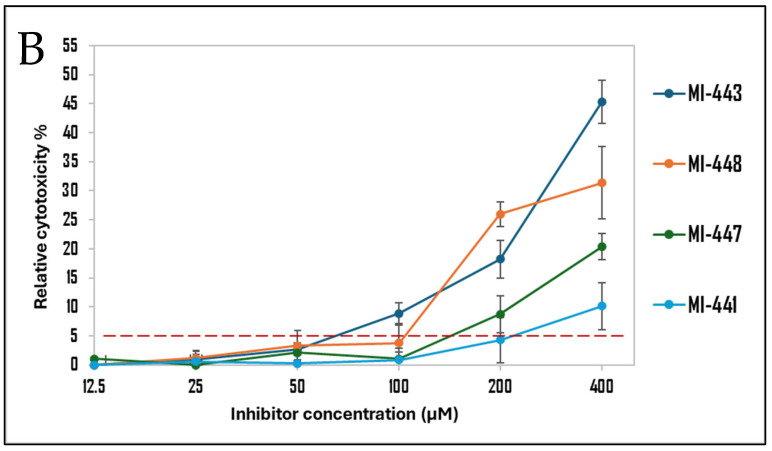
(**A**): The *in vitro* antiviral activity of TMPRSS2/matriptase inhibitors at a concentration of 50 µM on H1N1 replication in MDCK-II cells. The endpoint titration results (left vertical axis): black dots—individual data; bars—mean ± S.E.M. The asterisks indicate a significant reduction in the virus titers compared to the virus control by Welch’s *t*-test; * *p* < 0.05. The estimated cytopathic effect (CPE in %) is indicated on the right axis by separate ◊ markers. The microscopic images are example viewfields of different experimental groups (200× magnification). From left to right: negative control, virus control, and MI-441; MI-447. (**B**): The cytotoxicity assay of TMPRSS2/matriptase inhibitors in MDCK-II cells. The extracellular lactate dehydrogenase activity released by damaged cells was measured by a colorimetric reaction. The relative cytotoxicity % was calculated from the absorbance (A492–A690 nm) values as follows: [experimental value − low control]/[high control − low control], where the “low control” is the LDH activity that is released from the untreated cells, while the “high control” is the maximum possible LDH activity (released from the lysed cells). The inhibitor concentrations causing <5% relative cytotoxicity (red interrupted line) were considered non-cytotoxic. Mean ± S.E.M. (n = 3).

The antiviral assay of the TMPRSS2/matriptase inhibitors was preceded by a cytotoxicity test of the compounds using a commercial kit (Cytotoxicity Detection Kit Plus (LDH)). Based on the results in MDCK-II cells, the inhibitors were used at 50 µM in the antiviral assay, which was the highest concentration at which none of the compounds showed notable (>5%) cytotoxicity (Figure 6B).

All four of the investigated inhibitors suppressed influenza virus multiplication in the MDCK-II cell line at 50 µM, which was clearly observable by the lower severity of the cytopathic effects after H1N1 infection of the cell cultures compared to the untreated virus control wells. At 96 h after the virus inoculation, the estimated ratio of cells showing cytopathic changes in the virus control was found to be 90% (mean of three parallel wells that were assessed by two experienced researchers), while the effect was reduced to a level of 35–45% in the inhibitor-treated wells of the experimental plate.

Although those were only semiquantitative results, the virus titration from the collected cell culture supernatants proved the inhibitors’ protective effect against H1N1 replication: the viral infectious titers were lower in the presence of the inhibitors (Figure 6A). Without the inhibitors (no treatment or vehicle only), 10^4.55^ TCID_50_/mL was measured, whereas titers in the supernatants of the treated MDKC-II cell cultures were found to be 10^3.56^ (MI-441), 10^3.41^ (MI-443), 10^3.71^ (MI-447), and 10^4.01^ (MI-448) TCID_50_/mL, respectively. The antiviral effect was significant for MI-441 (*p* = 0.0197), MI-443 (*p* = 0.0196), and MI-447 (*p* = 0.0368) but not for MI-448. Thus, according to these data, the inhibitor MI-443 has the highest antiviral potential of the tested inhibitors; however, the results of analogs MI-441 and MI-447 are very similar.

## 3. Discussion

Type II transmembrane serine proteases are critically involved in the proteolytic activation of viral surface glycoproteins such as influenza hemagglutinin and the SARS-CoV-2 S protein, thereby contributing to viral entry and fusion with host cells [9,11,12,23,33].

The pharmacological targeting of the TTSP function has been extensively investigated with the application of small-molecule, peptide-based, and peptidomimetic inhibitors since these host proteases serve as stable antiviral targets that are suitable for potential combination treatments at a minimal risk for resistance development. The covalent-binding small molecule serine protease inhibitors, including camostat, which was originally developed for the treatment of chronic pancreatitis and the anticoagulant nafamostat mesylate, both of which bind to the S1-pocket of trypsin-like serine proteases, have shown broad efficacy against influenza and human coronaviruses *in vitro* and in mouse models [34,35,36,37].

The inhibitors investigated in the present study belong to the Phe(3-Am)-based peptidomimetic inhibitor class targeting TTSPs, representing an alternative scaffold to previously reported KBT-based TMPRSS2 inhibitors, which have demonstrated potent antiviral activity against SARS-CoV-2 and certain influenza subtypes (e.g., [26,27,30,38,39,40]).

In a previous report, the numerous tertiary amide forms of sulfonylated Phe(3-Am)-derivatives were described, demonstrating relatively low K_i_ values against matriptase while preserving a strong selectivity against thrombin and fXa [41]. Later, Pilgram et al. designed a panel of 23 analogs optimized for enhanced specificity. Notably, inhibitor MI-463 (Figure S2) and related compounds that inhibited H9N2 influenza A replication in MDCK II cells, emphasizing the potential of inhibiting host cell serine proteases leading to antiviral effects [30].

A range of *in vitro* methods has been developed to predict the pharmaco-toxicological properties of Phe(3-Am)-derived host protease inhibitors in preclinical studies without the need for a preliminary animal sacrifice. *In vitro* safety assessments using non-tumorigenic human intestinal epithelial cells and primary human hepatocytes (PHHs) revealed an excellent tolerability of several structurally related Phe(3-Am)-derivatives, such as inhibitors MI-1900 and MI-1907 (Figure S2), at concentrations up to 50 µM [42]. These findings are in good agreement with our current results in MDCK-II cells. The present study confirms that Phe(3-Am)-based inhibitors can be safely employed as TMPRSS2/matriptase inhibitors against H1N1 influenza viruses. Notably, compounds MI-441, MI-443, and MI-447 demonstrated significant antiviral activity without any detectable toxicity at 50 µM.

The role of the inhibitors in the reduction in H1N1 multiplication might be attributed to suppressing the proteolytic function of TMPRSS2 and/or matriptase. The inhibitor MI-441 had the highest affinity to both transmembrane serine proteases among the tested compounds. Analogs MI-443 and MI-447 had comparable *in vitro* anti-influenza activity, despite having notably higher Ki values for TMPRSS2 inhibition. However, they effectively bind to matriptase, which also plays a crucial role in the proteolysis of certain HA proteins, facilitating viral entry into host cells. However, elucidating the exact impact of the individual host protease activities on the demonstrated antiviral potency requires further studies.

In the present study, the impact of the applied Phe(3-Am)-derived protease inhibitors on CYP3A was evaluated using human liver microsomes. Previously, it was found that structurally related inhibitors demonstrated potent CYP3A4 inhibition at nanomolar to low micromolar concentrations in various model systems involving human microsomes and recombinant isoenzymes, in addition to 2D and 3D primary human hepatocytes [31,43,44]. The structural features, such as the nature of the N-terminal biphenyl sulfonyl substitution pattern and differences in the used C-terminal tertiary amide groups (with aminopiperidide or with piperazide/piperidide moieties, partially substituted with various ureido groups alkylated with cyclohexyl and tert-butyl groups), were found to play a role in CYP3A4 modulation. The structural modeling suggested this is due to an electrostatic repulsion between the compound’s high positive charge and the key residues, such as Arg105, in the enzyme’s active site, which is preventing effective orthosteric binding [31]. Our findings show that the inhibitors MI-448 and MI-447 have the least CYP3A inhibitory effects out of all the tested concentrations (10–50 µM) among the applied compounds. Both of them have a monochloro substitution in the N-terminal biphenyl ring in addition to their C-terminal N-(2-aminoethyl)piperidide group.

Hammami et al. found that despite the structural similarity among MI-432 (compound #11) containing an *N*-terminal 2′,4′-dichloro-biphenyl-sulfonyl group (Figure S2), its analog MI-441 (compound #13 with a 2′,3′-dichloro-biphenyl-sulfonyl moiety), and inhibitor MI-443 (compound #15 containing a 3′,4′-dichloro-biphenylsulfonyl residue), all of which contain an N-terminal dichloro-substituted biphenylsulfonyl group, notable differences could be observed in their inhibitory profiles. Compounds MI-441 and MI-443 showed higher K_i_ values for matriptase (K_i_ = 11–18 nM) compared with compound MI-432 (K_i_ = 2 nM), whereas their inhibitory potency toward thrombin was comparable, with the inhibition constants in the range of 20–30 nM. In contrast, the inhibitor MI-432 displayed almost a ten-fold higher affinity for fXa than analogs MI-441 (K_i_ = 580 nM) or MI-443 (K_i_ = 370 nM) [29].

The monochloro-substituted compounds MI-447 and MI-448 are derived from a non-substituted biphenyl-3-sulfonyl scaffold (compound #8, Figure S2) first described by Steinmetzer et al. [28]. These compounds showed a comparable inhibitory potency toward matriptase (K_i_ = 26–29 nM) and thrombin (K_i_ ~ 15 nM). The inhibitor MI-447 demonstrated a higher affinity for TMPRSS2 compared to the analog MI-448. However, these K_i_ values remain relatively high in contrast to their strong inhibitory impact on thrombin, which may increase the potential risk of bleeding side effects. Although the low K_i_ values observed for thrombin can be associated with coagulation-related adverse effects, the affinity toward fXa was comparatively weak (K_i_ values ~450–570 nM).

It can be stated that dibasic inhibitors containing a C-terminal 2-aminoethylpiperidide moiety and a (di)chloro substitution pattern on the N-terminal ring of the biphenyl group possess a significant antiviral potency, which is characterized by low inhibitory constants for matriptase and/or TMPRSS2 and higher K_i_ values for fXa. The binding mode of MI-441 (the most potent representative of the series) with matriptase and TMPRSS2 was modeled with Chai-1, a state-of-the-art co-folding model: MI-441 was placed in the respective active sites in the “canonical” binding mode of related protease inhibitors, displaying a salt bridge with the Asp189 residue in the S1 specificity pocket, with additional stabilizing H-bonds to neighboring residues, including Gly216 and Gly219. However, low K_i_ values for thrombin might indicate potential adverse effects leading to bleeding complications.

Since inhibitors MI-441, MI-443, MI-447 and MI-448 did not influence the fluorescence emission signal of HSA, it is reasonable to assume that they do not form high-affinity complexes with the abundant plasma protein. These results are in accordance with the earlier observations regarding several structurally related Phe(3-Am)-derived inhibitors [43,44]. However, fluorescence spectroscopic measurements highlighted that compounds MI-441, MI-443, MI-447, and MI-448 interact with AGP (Figure 5). The *K_a_* values of MI-441–AGP and MI-447–AGP complexes were around 6 to 8 × 10^5^ L/mol, suggesting strong interactions between the inhibitors and this protein. In our recent report, the binding constants that were determined with fluorescence quenching and ultracentrifugation techniques showed good correlations for ligand–AGP complexes [45]. Furthermore, a previous study also indicated the strong interactions of some Phe(3-Am)-type inhibitors (MI-472 and MI-477; Figure S2) with AGP [44]. Our results suggest the possible importance of AGP in the plasma protein binding of inhibitors MI-441, MI-443, MI-447 and MI-448.

In conclusion, TTSPs such as TMPRSS2 and matriptase represent validated and highly druggable host factors that are required for the activation of certain respiratory viruses. The peptidomimetic inhibitors studied in this research that simultaneously block TMPRSS2 and matriptase activity offer a potential antiviral strategy against pandemic-prone strains such as H1N1, H7N9, and H9N2. A continued exploration of structure–activity relationships, inhalation formulation strategies, host–pathogen dynamics, and improving the compounds’ selectivity will be essential to fully realize the therapeutic potential of the inhibition of host serine proteases.

## 4. Materials and Methods

### 4.1. Preparation of Inhibitor Solutions

The inhibitors were prepared as stock solutions in dimethyl sulfoxide (DMSO, Merck, Darmstadt, Germany) at a concentration of 10 mM and stored at −20 °C. The working solutions were freshly prepared by diluting the stock solutions with buffer solution or with the cell maintenance medium immediately prior to use [44]. The chemical structures of the compounds are presented in Figure 1. For CYP3A enzymatic assays, inhibitor concentrations of 10, 25, and 50 µM were employed, while a 50 µM concentration was used for the antiviral activity assay. Following the treatment with the inhibitors at 37 °C (with untreated samples serving as controls), the samples were analyzed for cytotoxicity, antiviral efficacy, enzyme kinetics, protein binding, and microsomal CYP3A activity.

### 4.2. Determination of Inhibition Constant for TMPRSS2

Soluble TMPRSS2 was prepared as described previously [33]. The kinetic measurements of TMPRSS2 were taken at room temperature in black 96-well microplates (Nunc, Thermo Fisher Scientific, Waltham, MA, USA) using a Tecan Spark^®^ multimode microplate reader (Tecan Group AG, Männedorf, Switzerland). The fluorescence was monitored with excitation at 380 nm and emission at 460 nm. For the TMPRSS2 assays, each well contained 100 µL of buffer containing the inhibitor (or without the inhibitor as a control), 20 µL of substrate solution (100 µM in assay, dissolved in water), and reactions were initiated by the addition of 20 µL TMPRSS2 solution (42.6 pM in assay), yielding a total assay volume of 140 µL [44]. The K_i_ values of these inhibitors for matriptase, thrombin, and factor Xa were taken from previous studies.

### 4.3. Cell Line for Virus Propagation

The Madin–Darby canine kidney II (MDCK-II) cells (Cytion, Eppenheim, Germany) were cultured using Dulbecco’s Modified Eagle Medium (DMEM) (Capricorn Scientific, Ebsdorfergrund, Germany) and were supplemented with 5% fetal calf serum (FCS), penicillin, streptomycin, and amphotericin B (Merck, Darmstadt, Germany). The cells were grown and maintained in a humidified incubator at 37 °C and with 5% CO_2_.

The MDCK-II cell line was used for the propagation of the H1N1 influenza virus (A/Puerto Rico/8/1934 strain). As an infection medium, FBS-free DMEM was supplemented with tosyl phenylalanyl chloromethyl ketone (TPCK)-treated trypsin (1 μg/mL; Merck, Darmstadt, Germany) along with the antibiotics and antimycotics. Following 5 days of incubation, the culture supernatant was collected, and the cellular debris was removed by low-speed centrifugation before subsequently storing (−80 °C).

### 4.4. In Vitro Evaluation of Antiviral Activity

The antiviral assay was performed on the H1N1-infected MDCK-II cells and treated with TMPRSS2/matriptase inhibitors. The cell suspension was grown in 96-well microplates (TPP, Trasadingen, Switzerland) and incubated until the monolayers reached 85–95% confluency (typically at 48 h). Then, 10^2.5^ TCID_50_ H1N1 virus suspension was inoculated into each well using an infection medium (except the uninfected cell control wells), infecting cells at a multiplicity of infection (MOI) of 0.005.

Prior to the antiviral experiment, the compounds were tested for cytotoxicity using a commercial kit that detects extracellular lactate–dehydrogenase activity to assess cellular damage (Cytotoxicity Detection Kit Plus (LDH); Roche, Basel, Switzerland) on the MDCK-II cells. The antiviral compounds were added to three parallel wells after 1 h of incubation with the virus suspension. Along with the inhibitor-treated wells, virus control (without inhibitors), vehicle control (0.5 *v*/*v*% of DMSO), and cell control (culture medium only), the wells were set up on each microplate. The monolayers were observed using an inverted microscope every day for the presence of H1N1-induced cytopathogenic effects (CPE). These included multifocal cell rounding, later followed by the detachment of affected cells from the wells’ surface and a mild to moderate vacuolization in the cytoplasm. The results were assessed at 96 h post-infection, as the CPE formation was the most pronounced at this time point.

The initial semiquantitative evaluation of antiviral activity was carried out by the microscopic observation of each well of the test plates by two independent operators, estimating the percentage of cells showing CPE. For the quantification of the inhibitory effect of TMPRSS2 inhibitors on influenza virus replication, the supernatants were collected from the cell cultures. The H1N1 infectious titers were determined by an endpoint titration assay: the supernatants were titrated in the MDCK-II cells in quadruplicates. The titer calculations were based on the Spearman–Kärber formula [46,47] after a 96 h incubation time.

### 4.5. Evaluation of CYP3A Inhibitory Activity

All the experiments were conducted using pooled human hepatic microsomes (50 donors, Gibco, Biocenter, Szeged, Hungary), containing a representative mixture of CYP enzymes, including CYP3A4 and CYP3A5 activities. The protein concentrations were quantified with the BCA Protein Assay Kit (Pierce, Thermo Fisher Scientific, Waltham, MA, USA), and 100 µg of microsomal protein was applied per assay well. The CYP3A enzymatic activity was evaluated by employing commercial assay kits (BioVision, Inc., Kampenhout, Belgium). For microsomal preparations, 30 µL of microsomes (20 mg/mL) were combined with 50 µL of an NADPH-generating system in an assay buffer [31].

The aliquots of the microsomal suspension (50 µL) were incubated with 20 µL of either the reference CYP3A inhibitor ketoconazole (KCZ; positive control) or the protease inhibitors at concentrations of 10, 25, or 50 µM for 30 min. The control wells received 20 µL of inhibitor-free buffer, whereas the background controls contained 70 µL of an assay buffer without microsomes or inhibitors. The reactions were initiated by the addition of an assay buffer containing the NADPH-generating system to the microsome–inhibitor mixtures. After a 15 min preincubation at 37 °C, 30 µL of the CYP3A substrate/NADPH mixture was added, yielding a final reaction volume of 100 µL. The CYP3A enzyme activity was quantified fluorometrically on a Victor X2 2030 reader (PerkinElmer, Waltham, MA, USA) at excitation/emission wavelengths of 535/587 nm.

### 4.6. Molecular Modeling

The binding poses for MI-441 were predicted with the state-of-the-art co-folding model, Chai-1 [32], using the respective protease domain sequences (matriptase: residues 16–244 from Uniprot entry Q9Y5Y6, TMPRSS2: residues 148–491 from Uniprot entry O15393) and the SMILES representation of MI-441 as input. PyMOL was used for visualization (the PyMOL Molecular Graphics System, Version 3.0, Schrödinger, LLC., New York, NJ, USA). To refer to residue positions, chymotrypsinogen numbering is used throughout this manuscript.

### 4.7. Spectroscopic Studies

The interactions of inhibitors MI-441, MI-443, MI-447, and MI-448 with the HSA and AGP were evaluated based on the fluorescence quenching experiments [44,45]. The absorption and fluorescence emission spectra were collected by applying a U-3900 UV-Vis spectrophotometer and a F-4500 fluorometer, respectively (Hitachi, Tokyo, Japan). Increasing concentrations of the inhibitors (0–10 μM) were added to the HSA (2 μM; λ_ex_ = 295 nm) or AGP (2 μM; λ_ex_ = 285 nm) in phosphate-buffered saline (PBS, pH 7.4). The fluorescence emission spectra were recorded, and after the background and inner-filter effect corrections [45], the data were evaluated at 340 nm and 335 nm for the HSA and AGP, respectively. The binding/association constants (*K_a_*) of the MI-441–AGP and MI-447–AGP complexes were determined with the HypSpec 2014 program (Protonic Software, Leeds, UK), as it has been described previously [48].

### 4.8. Statistical Analysis

The statistical analyses were conducted using R software (version 4.5.0, 2025, Vienna, Austria; https://cran.r-project.org/bin/windows/base/old/4.5.0/ (accessed on 13 August 2025)). The differences among the experimental groups were assessed with a one-way analysis of variance (ANOVA) followed by Tukey’s post hoc test for multiple comparisons. The data sets were checked for normality prior to the analysis. The results are presented as mean ± standard deviation (SD) or standard error of the mean (SEM). The statistical significance was defined as *p* < 0.05 (*), *p* < 0.01 (**), and *p* < 0.001 (***). All the experiments were independently repeated at least three times.

## Figures and Tables

**Table 1:** The inhibition constants (K_i_ ± SD in nM) of the tested compounds. The index (a) indicates K_i_ values reported in previous studies (MI-441 and MI-443 [29] and MI-447 and MI-448 [28]).

K_i_ Values (nM)				
**Inhibitors**	MATRIPTASE	B-THROMBIN	FACTOR XA	TMPRSS2
MI-441	11 ^(a)^ ± 2.7	20 ^(a)^ ± 0.05	576 ^(a)^ ± 2.3	63 ± 5
MI-443	18 ^(a)^ ± 1.1	29 ^(a)^ ± 0.25	374 ^(a)^ ± 1.95	224 ± 14
MI-447	29 ^(a)^ ± 2.1	14 ± 1.5	267 ± 4.5	192 ± 35
MI-448	26 ^(a)^ ± 0.4	15 ± 1.9	473 ± 19.6	396 ± 34

## Data Availability

All the raw data supporting the results of the present study can be obtained from the corresponding author upon reasonable request.

## Supplementary Materials

The following supporting information can be downloaded at https://www.mdpi.com/article/10.3390/antibiotics15040366/s1. Figure S1: The absorption and fluorescence emission spectra of MI-441, MI-443, MI-447, and MI-448, and the effects of MI-443 and MI-448 on the emission signal of AGP. Figure S2: The chemical structures of the inhibitor compounds in the Section 3.

## Abbreviations

ACE2	angiotensin-converting enzyme 2
AGP	α_1_-acid glycoprotein
CPE	cytopathic effect
CYP3A	cytochrome P450 3A
DMSO	dimethyl sulfoxide
fXa	factor Xa
HA	hemagglutinin
HAT	human airway trypsin-like protease
HSA	human serum albumin
KBT	ketobenzothiazole
KCZ	ketoconazole
KO	knockout
MDCK-II	Madin–Darby canine kidney II cells
MERS-CoV	Middle East respiratory syndrome coronavirus
PD	pharmacodynamics
Phe(3-Am)	3-amidinophenylalanine
PHH	primary human hepatocyte
PK	pharmacokinetics
SARS-CoV	severe acute respiratory syndrome coronavirus
TMPRSS2	transmembrane protease, serine 2
TTSPs	type II transmembrane serine proteases
WT	wild type
