# Supplementary material for: 3-Amidinophenylalanine-Derived Inhibitors’ Antiviral Effect Against H1N1 Influenza A Virus
Source: Antibiotics (Basel). 2026 Apr 2;15(4):366. doi: 10.3390/antibiotics15040366 (PMC13114104; doi:10.3390/antibiotics15040366)
Supplement: Supplementary file 1 [file antibiotics-15-00366-s001.zip › antibiotics-4203308-supplementary.pdf]

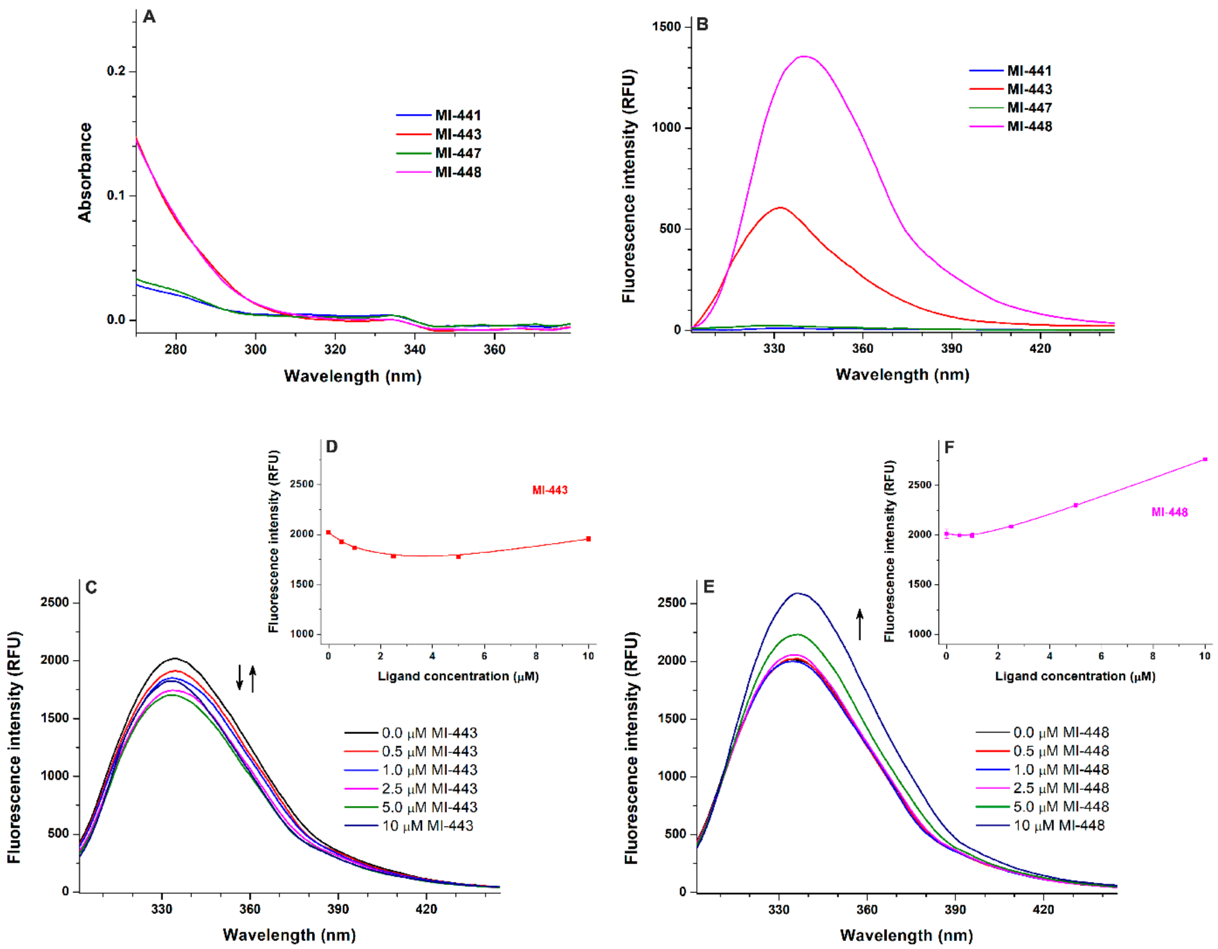

Figure S1: Fluorescence spectroscopic results. Absorption spectra of MI-441, MI-443, MI-447, and MI-448 (each 10 μM) in PBS (**A**). Fluorescence background signals of MI-441, MI-443, MI-447, and MI-448 (**B**; each 10 μM) in PBS (λ_{ex} = 295 nm). Fluorescence emission spectrum of AGP (2 μM) in the presence of increasing concentrations (0–10 μM) of MI-443 (**C**) and MI-448 (**E**). Concentration-dependent changes in the fluorescence emission intensity at 335 nm in the presence of MI-443 (**D**) and MI-448 (**F**) (the background and the inner-filter effect have been corrected).

#8

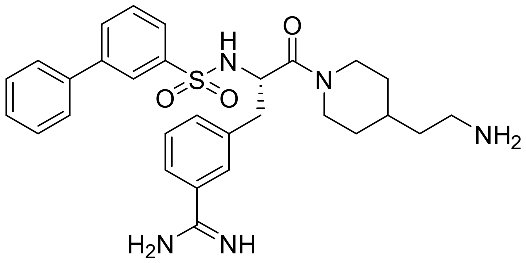

MI-432 (#11)

MI-463

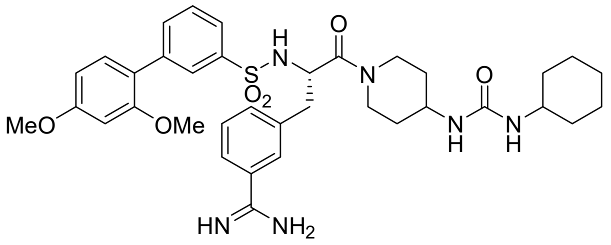

MI-472

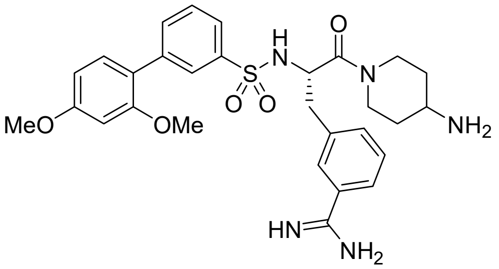

MI-477

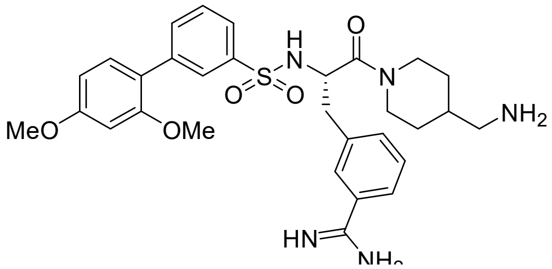

MI-1900

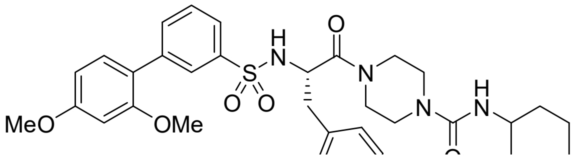

MI-1907

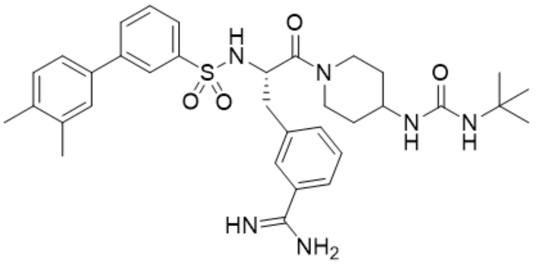

Figure S2: Chemical structures of the inhibitor compounds in the Section 3. Structures of MI-463 (Pilgram et al. 2022); MI-1900 and MI-1907 (Hammami et al. 2012; Pászti-Gere et al. 2021); compound #11 (Hammami et al. 2012) and compound #8 (Steinmetzer et al. 2009); MI-472 and MI-477 (Pilgram et al. 2022; van Eijk et al. 2023).
